# Assessing social cognition and risk-taking behaviour in patients with young-onset dementia: Study protocol for the YOD-RiSoCo observational prospective cohort study

**DOI:** 10.1371/journal.pone.0324517

**Published:** 2025-05-27

**Authors:** Floor Gelmers, Rients B. Huitema, Myrthe E. Scheenen, Barbara C. van Munster, Jacoba M. Spikman, on behalf of the YOD-INCLUDED Consortium

**Affiliations:** 1 Alzheimer Center Groningen, University Medical Center Groningen, Groningen, The Netherlands; 2 Department of Neurology, Unit Neuropsychology, University Medical Center Groningen, University of Groningen, Groningen, The Netherlands; 3 Department of Geriatrics, Martini Hospital, Groningen, The Netherlands; PLoS ONE, UNITED STATES OF AMERICA

## Abstract

**Background:**

Certain subtypes of young onset dementia (YOD), such as the behavioural variant of FTD or the behavioural variant of AD (bvYOD), present with changes in social behaviour instead of memory impairments. These symptoms are often under-recognized, delaying the diagnosis and contributing to psychosocial problems. Impairments in social cognition (SC), an important affected domain in bvYOD, underlie these social behavioural changes. Especially emotional blunting and a lack of empathy in patients with bvYOD might be related to problematic social behaviour, such as risk-taking behaviour, which may potentially harm others. However, despite the importance of SC impairments in the diagnosis of YOD and the impact of SC impairments on social behaviour, there is a lack of valid and well normed measures for certain aspects of SC, such as emotion experience and empathy.

**Methods:**

The YOD-RiSoCo study is an observational prospective cohort study, consisting of two separate, but related, studies. Study 1 includes 64 patients with bvYOD and 64 healthy controls to assess the sensitivity and validity of newly developed SC instruments for measuring emotion experience and empathy, by comparing their average group performance. Furthermore, validity of the new instruments will be assessed by analysing the associations of performances on these new tests with those on more traditional SC and other neurocognitive tests. Study 2 focuses on assessing to which extent SC measures relate to risk-taking behaviour. This study includes 20 patients with bvYOD and 20 healthy controls from Study 1, in addition to 20 patients with non-bvYOD (e.g. Alzheimer’s dementia or vascular dementia) and 20 patients with serious brain injury affecting frontal networks. A specific question is whether the relationship between SC and risk-taking behaviour is generic (for all groups with SC impairments), or specific (not in dementia without SC impairments).

**Discussion:**

Results of the YOD-RiSoCo study will yield new, sensitive neuropsychological tests for aspect of social cognition, which may contribute to a more timely diagnosis of YOD, allowing earlier provision of appropriate counselling and care for patients and their close others. Furthermore, the study will contribute to a better identification of those social behavioural symptoms that negatively affect functioning and social relations.

**Trial registration:**

The trial is registered at www.clinicaltrials.gov with identifier NCT06286293

## Background

Young-onset Dementia (YOD) refers to dementia with the onset before the age of 65 [1,2]. The global age-standardized prevalence of YOD is approximately 119 per 100.000 population [2]. Alzheimer’s disease (AD), vascular dementia (VD), and frontotemporal dementia (FTD) are the dominant causes of YOD, including various different subtypes as well [2,3]. Characteristic of YOD, other than in later-onset dementia, is that memory impairments are not predominant, and may not be present until later in the disease process [4]. In some variants of YOD, such as the behavioural variant of frontotemporal dementia or the behavioural variant of Alzheimer’s dementia, YOD can present in its early stages with social behavioural changes. These social behavioural changes can include behaviours that have a negative impact on other people, or can even endanger them, such as disinhibition, aggression and risk-taking behaviour. Symptoms in the early disease stages of YOD, especially changes in social behaviour, are often under-recognized, which delays the diagnosis and often contributes to psychosocial problems [5–7].

Related to these behavioural changes is social cognition (SC), an important affected domain in the behavioural variants of YOD (bvYOD), which underlies the abilities to exhibit appropriate social behaviour. Social cognition concerns the ability to perceive socially relevant information, understand the thoughts, feelings and intentions of others and respond adequately [8]. Important aspects of SC are the recognition of facial expressions of emotions, perspective taking (also referred to as mentalizing or Theory of Mind [ToM]), and empathy. ToM can be divided into cognitive ToM, concerning the ability to assess another’s thoughts and knowledge, and affective ToM, concerning the ability to assess another’s feelings and emotions through cognitive processes [9]. Empathy on the other hand, is a more emotional process of sharing in another’s affective state and feeling with another’s emotions [9].

In patients with bvYOD, impairments can occur in all these aspects of SC, and especially emotional blunting and a lack of empathy are often reported by close others [10]. This lack of emotion experience and empathy might be related to problematic behaviour, such as risk-taking behaviour, as patients do not experience the signalling function of emotions, such as fear, to warn them for possible risks [11,12]. Furthermore they might also be unable to sufficiently experience the emotional impact of their actions on other people [11,12]. There is already some evidence of a relationship between SC aspects, including empathy, and problematic social behaviour, such as risk-taking behaviour [13–15]. However, studies into the relationship between empathy and risk-taking behaviour assess empathy with subjective questionnaires, or indirect measures, such as (functional) imaging data, for empathy, instead of performance-based measures.

Despite the importance of SC in the diagnosis of YOD and the impact of SC impairments on social behaviour, such as risk-taking behaviour, there is a lack of sensitive and valid neuropsychological tests for measuring SC aspects. To date, only tests for emotion recognition, and some ToM tests are validated and widely used. No valid performance-based instruments exist to measure the ability to experience emotions and to feel with another (empathy).

In conclusion, we state that 1) people with bvYOD often present with problems in social cognition and social behaviour, 2) impairments in different aspects of social cognition (emotion recognition, ToM, emotion experience and empathy) are related to problematic social behaviour, such as risk-taking behaviour, and 3) to date there is a lack of valid and reliable performance-based measures for these aspects of social cognition, in particular empathy and emotion experience, that are also predictive of such negative social behaviours. Therefore, the main aims of the YOD-RiSoCo study are the following:

1) Assess sensitivity of new performance-based SC measures (including emotion experience and empathy) ◊ Study 1
2) Assess construct validity of the new performance based SC measures ◊ Study 1
3) Assess the relationship between social cognition and unsafe risk-taking behaviour (measuring ecological validity of the new SC tests), and investigate if this relationship is generic (by including patients with brain injury affecting frontal networks) or specific (by including patients with non-bvYOD variants, for whom no SC impairments or risk-taking behaviour are expected) ◊ Study 2

As a result of this study, we hope to introduce new, sensitive neuropsychological instruments which may aid in a more timely diagnosis of bvYOD and a better identification of social behavioural symptoms in general.

## Methods and materials

### Study design study 1

Study 1 of the YOD-RiSoCo study is an observational prospective cohort study, that aims to include 64 patients with bvYOD and 64 healthy controls. Controls will be matched to patients on a group level, so that sex, age, and educational level distributions do not significantly differ between groups. The recruitment of controls will be adapted to match the included patients.

### Study design study 2

**Fig 1:**
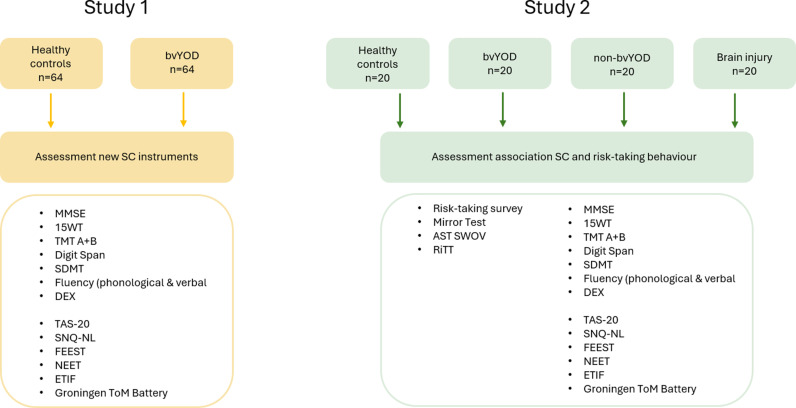
Flow-chart study design.

Study 2 of the YOD-RiSoCo study is an observational study that aims to include 20 patients with bvYOD, 20 healthy controls, 20 non-bvYOD patients and 20 patients with brain injury affecting frontal processes. The bvYOD and healthy control patients will be included from Study 1 (Fig 1).

### Study population

#### Inclusion and exclusion criteria Study 1.

All participants must have sufficient command of the Dutch language and must be between 18 years and 70 years of age. Patients in the bvYOD group must have a probable diagnosis of young-onset (start of symptoms before 65 years old) bvFTD according to the current criteria [16], or bvAD according to current criteria [17,18], or another YOD subtype in which social behavioural changes are prominent, confirmed after interdisciplinary consensus meeting in which interviews, neuropsychological examination, neurological and psychiatric assessments, neuro-imaging, blood samples, and in some cases FDG/PIB-PET-scans, CSF biomarkers, or genetic counselling were discussed. Furthermore, patients must still have the capacity to consent to participate in the study, and be able to undergo a neuropsychological assessment. Capacity to consent was determined by a Clinical Neuropsychologist, based on the neuropsychological assessment. This consent procedure is approved by the ethics committee of the University Medical Center Groningen.

Exclusion criteria for bvYOD are presence of (premorbid) severe neurological or psychiatric pathology (e.g., severe depression, schizophrenia, substance abuse) non-related to dementia. For healthy controls the exclusion criteria are presence of serious psychiatric disorders or a history of neurological disorders, which may interfere with cognitive functioning (e.g., recent concussion, previous subarachnoid or intracerebral haemorrhage, intracranial tumours, epilepsy or ischemic stroke).

#### Inclusion and exclusion criteria Study 2.

All participants must have sufficient command of the Dutch language, be between 18 years and 70 years of age, and have any driving experience in the past five years. Non-bvYOD subjects are required to have a probable diagnosis of young-onset dementia (start of symptoms before 65 years old), other than a behavioural YOD subtype (such as bvFTD, or bvAD), for example amnestic variant AD (according to McKhann criteria[18]), confirmed after interdisciplinary consensus meeting. Inclusion criteria for patients with brain injury are a brain injury, such as moderate to severe traumatic brain injury, stroke, or a brain tumour, impacting frontal processes. Furthermore, all patients must still have the capacity to consent to participate in the study and be able to undergo a neuropsychological assessment. Capacity to consent was determined by a Clinical Neuropsychologist, based on the neuropsychological assessment. This consent procedure is approved by the ethics committee of the University Medical Center Groningen

Exclusion criteria for subjects with non-bvYOD or brain injury are presence of (premorbid) severe neurological or psychiatric pathology non-related to dementia or brain injury.

The in- and exclusion criteria described for bvYOD and healthy subjects in Study 1 also apply for bvYOD and healthy subjects participating in Study 2.

#### Recruitment.

Patients with YOD will be recruited from the memory clinic (Alzheimer’s centre) of the University Medical Center Groningen (UMCG), memory clinics of local regional hospitals, and other academic memory clinics that are part of YOD-INCLUDED. Patients with brain injury impacting frontal processes will be recruited from the department of Neurology, subdepartment Neuropsychology, of the UMCG. Healthy controls will be recruited through convenience sampling by distributing flyers with information about the study and a call to participate. In addition, patients with YOD and healthy close others can also be included through the JMD (‘*Jonge mensen met dementie en hun naasten’*, which is Dutch for ‘Young people with dementia and their close others’) study of YOD-INCLUDED. YOD-INCLUDED is a Dutch national consortium, which the YOD-RiSoCo study is part of, aimed at improving diagnoses and care for patients with YOD. The JMD study is a national cohort study, for which patients with YOD and their close others can sign themselves up. Patients with YOD and healthy close others, who take part in the JMD study, will be invited to participate by the JMD research assistant through e-mail. They can also apply themselves for the module “YOD RiSoCo” in their personal dashboard of the JMD study, at www.yod-incuded.nl/jongemensenmetdementie.

### Study procedures

Patients are provided with written information about the study by their clinician (e.g., geriatrician, neurologist, or (clinical) neuropsychologist), after diagnosis. This includes information about the study, participation, and an informed consent form, to read through at home. Two weeks after receiving the information and informed consent, patients are called by the researcher to make sure patients are fully informed and to create the possibility to ask questions regarding the study. If patients do not wish to be called, they can inform the investigator. When written informed consent is received, participants will be contacted by the researcher to schedule an appointment for the assessment. Data from tests that are administered to patients in a prior neuropsychological assessment will be obtained with the patient’s permission, if the neuropsychological assessment took place in the last 6 months (for patients with YOD) or two years (for patients with brain injury). These tests will then be omitted in the study assessment.

#### Study 1: Assessment procedure.

The assessment consists of several neuropsychological tests and questionnaires, and a short anamnesis concerning demographic characteristics (Fig 1). Some tasks are pencil-and-paper tasks, other tasks are computerized. The total duration of the assessment is 2.5 to 3 hours, including a short break.

#### Study 2: Assessment procedure.

Study 2 consists of the same assessments as study one and includes three additional neuropsychological tasks and a driving simulator task (Fig 1). The total duration of Study 2 is approximately 4.5 hours, including a lunch break.

### Data collection Instruments

#### General information.

Demographical characteristics (age, sex, and educational level) and data with regard to the diagnosis (e.g., imaging, biomarker and genetic data) will be collected to describe the included patient groups and to account for possible confounding factors.

#### Study 1: Assess sensitivity and validity of the new social cognition instruments.

To assess if the newly developed instruments can differentiate between the presence of frontal social cognitive pathology and the absence of such pathology, and to assess the construct validity of these new instruments, the newly developed instruments, as well as existing social cognition measures and general cognitive measures, will be administered to participants. See Table 1.

**Table 1:** Assessment instruments Study 1 New SC instruments.

Assessment	Construct
**General information**	
Demographics	Age, sex, educational level
Medical information	Diagnosis, time since diagnosis, dementia type (including genetics), imaging data (MRI, PET).
Driving experience questionnaire – self	Driving experience (e.g., age obtaining driving license, lifelong number of driven kilometres, driving behaviour)
Driving experience questionnaire – proxy	Proxy-rated driving behaviour
**New social cognition measures**	
Nummenmaa Emotion Experience Test (NEET)	Bodily experience of emotions
Empathy Task of Identifying emotions and Feeling with another (ETIF)	Empathy; identifying emotions and feeling with another
Groningen Theory of Mind Battery	Cognitive and affective Theory of Mind, verbally as well as non-verbally
**Existing social cognition measures**	
Facial Expressions of Emotions – Stimuli and Test (FEEST)	Facial emotion recognition
Social Norms Questionnaire (SNQ-NL)	Compliance with and understanding of social norms
Toronto Alexithymia Scale (TAS-20)	Alexithymia
**General cognitive measures**	
Mini Mental State Examination (MMSE)	Cognitive screener
15 Words Test (15WT)	Verbal memory
Trail Making Test (TMT)	Psychomotor speed, visual attention, and mental flexibility
Digit span	Working memory
Symbol Digit Modalities Test (SDMT)	Mental speed
Letter fluency	Language, phonological fluency, executive control
Verbal fluency	Language, semantic fluency
Dysexecutive Questionnaire (DEX) – self	Dysexecutive syndrome and behavioural changes, self-reported
Dysexecutive Questionnaire (DEX) – proxy	Dysexecutive syndrome and behavioural changes, proxy-reported

The Nummenmaa Emotion Experience Test (NEET) is designed to measure bodily experience of emotions, i.e., if someone is able to experience and identify in which parts of their body, they experience specific emotions. The concept of the test is derived from research of Lauri Nummenmaa [19] who constructed emotion-specific maps of bodily sensations, using a topographical self-report method. For the NEET, participants are presented with an empty map of a body, accompanied by a specific emotion. The test includes the following eleven emotions: anger, fear, disgust, happiness, sadness, surprise, love, contempt, pride, shame, and envy. Participants are instructed to take time to evoke this emotion within themselves, by thinking about a situation in which they have felt this emotion or would possibly feel this emotion. Then, they must click on the body parts in which they experience that specific emotion, resulting in the appearance of a red glow in that body part. The map includes the following body parts: 1) head (including face); 2) throat; 3) chest; 4) arms; 5) hands; 6); abdomen; 7) pelvic area; 8) legs; and 9) feet. Before starting the test, participants are provided with two practice items for the emotions ‘nervous’ and ‘disappointed’.

The Empathy Task of Identifying emotions and Feeling with another (ETIF) is inspired by the Empathic Accuracy Task paradigm of Zaki and colleagues [20]. To construct the ETIF, eleven male and female Dutch-speaking persons of different ages were recruited and asked if they would like to participate in the development of a new test for measuring empathy. Before filming these ‘targets’ were asked to recall a specific autobiographical event, in which they experienced one of the six basic emotions (i.e., happiness, fear, anger, surprise, disgust, or sadness). They were asked to write a short summary of each event they could recall, and to rate its overall emotional intensity on a 5-point scale, ranging from 1 (‘did not feel the emotion at all’) to 5 (‘experienced the emotion very strongly’). Only events with a rating of 4 or 5 were filmed. Before filming, targets were instructed to take their time recalling the situation to reinstate the affective state they had felt during the event. When talking about the situation in front of the camera, they were not allowed to make specific references to their affective state (i.e., ‘sad’ or ‘happy’), but they were allowed to describe their bodily symptoms (‘shaking hands’, ‘lump in my throat’). All targets were filmed from the upper chest upwards, in front of a white wall, for standardisation purposes. Each clip lasted between 16 seconds and 70 seconds (mean = 41.8, SD = 17.2). For each emotion, two clips were selected for the test, one including a woman and one including a man. During the administration of the ETIF, videos are presented full screen. After watching each video, participants are asked the following questions: 1) How do you think the person in the videoclip felt during the event? 2) How do you think the person in the videoclip felt while *talking about* the event? 3) Which of these emotions fits best to describe how the person in the videoclip felt while talking? Happy, surprised, fearful, angry, sad, disgusted, or neutral. 4) Does the videoclip evoke an emotion or a feeling in you? And if the participant answers this question with ‘yes’, it is asked 5) what emotion or feeling is that? With regards to scoring, only question 3 and question 5 are scored, with one point for a correct or appropriate answer. The answers to question 3 form the subscale ‘Identifying another’s emotions’, while the answers to question 5 form the subscale ‘Feeling with another’. Each subscale has a maximum score of 12, and both subscales combined form a total Empathy score.

The Groningen ToM battery consists of four parts including items of different already existing tests for measuring ToM. Of each included test, the six most ‘sensitive’ items were selected, based on which items showed the largest significant difference between a group of patients, and healthy controls. The first part of the Groningen ToM battery consists of six items of the Hinting Task [21]. A maximum of twelve points, two points per question, can be obtained on this part. Two points will be awarded if the participant immediately gives a correct answer, and one point will be awarded if the participant answers correctly after receiving a hint. No points will be awarded if the participant does not answer correctly. The second part of the Groningen ToM battery consists of six, slightly adapted, items of the Faux Pas [22–24]. The following questions are asked in the Faux Pas test: 1) Did anyone say something they should not have said or something awkward? If the participant answers with ‘yes’, question 2 and 3 are asked: 2) who said something they should not have said or something awkward? and 3) why should he/she not have said it or why was it awkward? Regardless of the answer on question 1, the following two questions are always asked: 4) How do you think (the person to whom something awkward was said) felt? and 5) How do you think (the person that said the awkward thing or something he/she should not have said, or a third person) felt? For this part, a maximum of twelve points can be obtained for correctly identifying whether there is a faux pas and why there is a faux pas, and a maximum of twelve points can also be obtained for correctly identifying how the persons in the stories feel. The third part of the battery includes three items of the Story-based Empathy Task (SET) [25] in which the ability to infer other’s intentions (cognitive ToM) is assessed, and three items of the SET in which the ability to infer other’s emotions (affective ToM) is assessed. Per item, participants are awarded one point for correctly describing how they think the story ends, and another point for choosing the right ending out of three options. Six points can be awarded for the cognitive ToM items, and six points can be awarded for the affective ToM items, resulting in a maximum of twelve points for this part. The last part of the Groningen ToM battery includes six adapted items of the Cartoon test [26]. The adaptations made to the Cartoon test are the addition of one or two extra questions as a subtle hint, and the use of a different scoring model. In the original version people are awarded 0–3 points per item, depending on the elements named in their answer. In our version, people are awarded two points for an answer that is immediately correct, and one point for an answer that is correct after the additional question(s) is/are asked. Zero points are awarded when someone is unable to give a correct answer. Participants can obtain a maximum score of twelve in total, including six points for ToM items, and six points for non-ToM items.

#### Study 2: Investigate relationship between social cognition and risk-taking behaviour (ecological validity).

To investigate the relationship between social cognition and risk-taking behaviour, new and existing social cognitive measures will be administered to participants. Furthermore, risk-taking behaviour will be assessed with the Action Selection Task (AST) from the Institute of Road Safety Research (SWOV), the newly developed Risk-Taking Survey, and the newly developed Risk-taking in Traffic Task (RiTT), see Table 2. To account for the influence of other cognitive aspects, all general cognitive measures from Study 1 will also be administered in Study 2.

**Table 2:** Assessment instruments Study 2 Association SC and risk-taking behaviour.

Assessment	Construct
Study 2 also includes all assessments that are conducted in Study 1	
Risk-taking in Traffic Task (RiTT) (new)	Risk-taking behaviour in a driving simulator task
Risk-taking survey (RTS) (new)	Risk-taking behaviour in daily life
Action Selection Task (AST)	Risk-taking behaviour in static traffic situations
Mirror Test	Mimicry

The Risk-taking survey measures the degree to which a person can accurately identify situations that contain a possible risk for themselves or for another person. It consists of 30 *Yes-No* questions to be completed by the participant after a standardized instruction by the examiner. The instruction is as follows: “Here you are provided with a list of behaviour of people. Is it in your opinion *dangerous* or *risky* to do these things? Please check yes or no. With regard to *risky*, think of something that has a considerable chance to have adverse consequences, immediately, or in the longer term, and what you, therefore, should better not do.” Examples of questions are *“Is it risky or dangerous to sit in the backseat of a car, without your seat belt fastened?”* (Yes), or *“Is it risky or dangerous to eat a pastry with whipped cream on a birthday, in the presence of young children?”* (No).

With the RiTT, risk-taking behaviour in traffic situations will be measured in a driving simulator, that is positioned on a moving platform. The simulator operates in automatic gearbox mode. First, the participant will complete two practice scenarios. The first practice scenario focuses on learning how to accelerate and how to manage the steering wheel. The scenario takes place in a rural environment, on a winding road. The second practice scenario is focused on learning how to adapt driving speed, and learning how to break, while also focusing on traffic and traffic rules. This again takes place in a rural environment, but now the participant will encounter cross-roads, for which different priority rules apply. If needed, one or both practice scenarios can be repeated. After the practice scenarios, the risk-taking scenario will be completed. In this scenario, the participant is driving through rural, but also urban, environments. As an instruction, the participant has been told that they are late for an appointment, and that they must get to their appointment as soon as possible. They are also told that they still must comply with traffic rules and that they should drive as they would drive in real life. During the route that the participant must drive, different situations occur in which there is a possible risk for the participant him-/herself, for others or for both self and others. There are also situations that are comparable to the risk situations, but do not include a risk.

### Data management

The data are stored in an electronic case report form (eCRF), using REDCap. Metadata are stored in the eCRF as well. Study monitoring is performed by in-house study monitors from the UMCG. Depending on the type of data and associated privacy regulations, data from the YOD-RiSoCo study will be made publicly available or will become available via the corresponding author, upon reasonable request.

### Statistical analysis

The analyses will be conducted in Statistical Package for the Social Sciences (SPSS). Assumptions for the intended analyses will be checked. Parametric tests will be used for normally distributed data, otherwise a non-parametric alternative will be used. For all analyses, the alpha will be set at 0.05, and analyses will be two-sided. In case of multiple comparisons, Bonferroni-Holm correction, or another appropriate correction, will be applied. Demographic variables will be analysed on a descriptive level (mean, standard deviation, frequency, percentage). To ensure matched groups of patients and controls, comparisons will be made on demographic variables with t-test (or non-parametric tests such as chi-square or Mann-Whitney U tests). Potential confounders will be included as covariates.

#### Study 1 New SC instruments.

Independent samples t-tests (or a non-parametric alternative, such as chi-square or Mann-Whitney U tests) will be performed to assess if the newly developed tasks can differentiate patients with bvYOD from healthy controls, based on social cognition, on a group level.

Construct validity of the newly developed measures will be assessed with parametric (such as Pearson’s correlation), or if more appropriate, non-parametric (such as Spearman’s) correlation analyses between the newly developed SC instruments, and existing SC instruments and other cognitive tests, respectively. It is expected that the new SC tests are significantly correlated to existing SC tests, indicating that the tests fall within the SC domain (convergent validity). However, no high correlation is expected, as the new tests measure different aspects of SC. No significant correlations are expected between the new SC tests and other cognitive measures (such as memory and attention), indicating that the tests measure SC specifically and no other cognitive domains, such as memory and attention (divergent validity).

#### Study 2 Relationship between social cognition and risk-taking behaviour (ecological validity).

The relationship between SC test performance and actual social behaviour (ecological validity), specifically risk-taking behaviour, will be investigated by analysing the relationship between the SC tests and the risk-taking measures with parametric or non-parametric correlation analyses in the different groups (bvYOD, non-bvYOD, brain injury, healthy controls). A significant correlation will demonstrate an association between social cognition and risk-taking behaviour and therefore, be indicative ecological validity. By looking specifically at the association between SC and risk-taking behaviour in patients with frontal brain injury, it will be assessed whether this relationship is generic for all patient groups with SC impairments. Furthermore, by assessing this relationship specifically in patients with non-bvYOD, for whom no SC impairments or risk-taking behaviour are expected, it will be assessed whether this relationship is specific.

### Sample size

The aim of the YOD-RiSoCo study is to aid in the diagnosis of YOD by improving measurement of social cognition. Therefore, the main outcome measure is the difference between bvYOD patients and healthy controls in performance on the new SC instruments. This way we hope to differentiate pathology in bvYOD from healthy controls and to prove the sensitivity of the instruments for the specific pathology.

The estimated effect size is based on a previous study assessing the sensitivity of a new social cognition task [27]. In this study, significant differences were found between patients and controls in performance on the SNQ-NL Total score, with a large effect size (η_p_^2^ = 0.23). However, considering that no studies have yet been conducted with the current measurements and in the current population, a moderate effect size of 0.5 (default option) for the power calculation was chosen.

The power calculation was performed with G*Power 3.1 [28]. For achieving 80% power, with 20% chance for making a Type II error and a 5% significance, the estimated sample size per group is 64. If the required number of approximately 128 inclusions is still not achieved within 24 months, the inclusion period will be extended with 6 months. In case of missing data, we will include additional subjects to reach the target N.

### Ethics approval and consent to participate

YOD-RiSoCo will be conducted according to the principles of the Declaration of Helsinki and the national and international standards of Good Clinical Practice. Potential participants receive detailed written and oral information on the study procedures and all participants will provide written informed consent. Ethical approval of the study protocol was obtained from the Medical Ethical Committee of the UMCG (2024/395). The YOD-RiSoCo study protocol is registered at clinicaltrials.gov, with identifier NCT06286293.

## Results

Recruitment started from January 2025 and is planned to continue until the end of 2026. The first publications are expected in early 2027.

## Discussion

To the best of our knowledge, the YOD-RiSoCo study will be the first to investigate the relationship between aspects of social cognition and risk-taking behaviour in a broad sample of patients with bvYOD, non-bvYOD, patients with brain injury affecting frontal networks, and healthy controls. Furthermore, we hope the study will lead to new valid instruments for measuring aspects of social cognition for which currently valid instruments are lacking.

First, the YOD-RiSoCo study will result in new sensitive and valid measures for aspects of SC, such as bodily emotion experience, and empathy. These measures will hopefully facilitate a better identification and deeper understanding of social cognitive difficulties in patients with behavioural variants of young-onset dementia. As patients with these bvYOD subtypes present with changes in social behaviour, related to SC impairments, the new instruments will hopefully lead to better recognition of these first symptoms, resulting in a timelier diagnosis.

Furthermore, by investigating the relationship between social cognition and risk-taking behaviour, the association between SC impairments and problematic social behaviour, such as risk-taking behaviour, will be assessed. Identifying risk-taking behaviour is important, as it can have detrimental consequences for patients themselves, but also for other people that might be unintentionally affected by risk-taking behaviour. If a relationship between SC and risk-taking behaviour is found in this study, guidelines regarding fitness to drive in patients with bvYOD or brain injury affecting frontal processes should be reviewed and revised to be more applicable to patients with SC impairments and social behavioural change.

In conclusion, the results of the YOD-RiSoCo study will contribute to a more comprehensive understanding of social cognition and risk-taking behaviour in patients with young-onset dementia, which may speed up the diagnostic process of young-onset dementia, allowing timely provision of appropriate counselling and care for patients and their close others.

## Supporting information

S1 Appendix List Consortium members. Full list of all YOD-INCLUDED consortium members. CSV S1 List consortium members YOD-INCLUDED.
(XLSX)

## References

[1] Koopmans R, Rosness T. Young onset dementia--what does the name imply?. Int Psychogeriatr. 2014;26(12):1931–3. doi: 10.1017/S1041610214001574 25382199

[2] Hendriks S, Peetoom K, Bakker C, Van Der Flier W, Papma J, Koopmans R. Global prevalence of young-onset dementia: A systematic review and meta-analysis. JAMA Neurol. 2021;78(9):1. 34279544 10.1001/jamaneurol.2021.2161 PMC8290331

[3] Vieira RT, Caixeta L, Machado S, Silva AC, Nardi AE, Arias-Carrión O, et al. Epidemiology of early-onset dementia: a review of the literature. Clin Pract Epidemiol Ment Health. 2013;9:88–95. doi: 10.2174/1745017901309010088 23878613 PMC3715758

[4] Kuruppu DK, Matthews BR. Young-onset dementia. Semin Neurol. 2013;33(4):365. 24234358 10.1055/s-0033-1359320 PMC4033406

[5] Van Vliet D, De Vugt M, Bakker C, Pijnenburg Y, Vernooij-Dassen M, Koopmans R. Time to diagnosis in young-onset dementia as compared with late-onset dementia. Psychol Med. 2013;43(2):423–32. 22640548 10.1017/S0033291712001122

[6] Woolley JD, Khan BK, Murthy NK, Miller BL, Rankin KP. The diagnostic challenge of psychiatric symptoms in neurodegenerative disease: rates of and risk factors for prior psychiatric diagnosis in patients with early neurodegenerative disease. J Clin Psychiatry. 2011;72(2):126–33. doi: 10.4088/JCP.10m06382oli 21382304 PMC3076589

[7] Mendez MF. The accurate diagnosis of early-onset dementia. Int J Psychiatry Med. 2006;36(4):401–12. doi: 10.2190/Q6J4-R143-P630-KW41 17407994

[8] Adolphs R. The neurobiology of social cognition. Curr Opin Neurobiol. 2001;11(2):231–9. doi: 10.1016/s0959-4388(00)00202-6 11301245

[9] de Waal FBM, Preston SD. Mammalian empathy: behavioural manifestations and neural basis. Nat Rev Neurosci. 2017;18(8):498–509. doi: 10.1038/nrn.2017.72 28655877

[10] Joshi A, Barsuglia J, Mather M, Jimenez E, Shapira J, Mendez M. Evaluation of emotional blunting in behavioral variant frontotemporal dementia compared to Alzheimer’s disease. Dement Geriatr Cogn Disord. 2014;38(0):79. 24603498 10.1159/000357838 PMC4104135

[11] Damasio A. Descartes’ error: emotion, reason and the human brain. Putnam Publishing. 1994.

[12] Bechara A, Damasio H, Damasio AR. Emotion, decision making and the orbitofrontal cortex. Cereb Cortex. 2000;10(3):295–307. doi: 10.1093/cercor/10.3.295 10731224

[13] Zelinková J, Shaw D, Mareček R, Mikl M, Urbánek T, Peterková L. Superior temporal sulcus and social cognition in dangerous drivers. Neuroimage. 2013;83:1024–30. 23911672 10.1016/j.neuroimage.2013.07.063

[14] Zelinková J, Shaw DJ, Mareček R, Mikl M, Urbánek T, Havlíčková D, et al. An evaluation of traffic-awareness campaign videos: empathy induction is associated with brain function within superior temporal sulcus. Behav Brain Funct. 2014;10:27. doi: 10.1186/1744-9081-10-27 25118071 PMC4149038

[15] van den Berg NS, Reesink FE, de Haan EHF, Kremer HPH, Spikman JM, Huitema RB. Emotion Recognition and Traffic-Related Risk-Taking Behavior in Patients with Neurodegenerative Diseases. J Int Neuropsychol Soc. 2021;27(2):136–45. doi: 10.1017/S1355617720000740 32812527

[16] Rascovsky K, Hodges JR, Knopman D, Mendez MF, Kramer JH, Neuhaus J, et al. Sensitivity of revised diagnostic criteria for the behavioural variant of frontotemporal dementia. Brain. 2011;134(Pt 9):2456–77. doi: 10.1093/brain/awr179 21810890 PMC3170532

[17] Dubois B, Villain N, Frisoni G, Rabinovici G, Sabbagh M, Cappa S. Clinical diagnosis of Alzheimer’s disease: recommendations of the International Working Group. Lancet Neurol. 2021;20(6):484–96. 33933186 10.1016/S1474-4422(21)00066-1 PMC8339877

[18] McKhann GM, Knopman DS, Chertkow H, Hyman BT, Jack CR, Kawas CH. The diagnosis of dementia due to Alzheimer’s disease: recommendations from the National Institute on Aging-Alzheimer’s Association workgroups on diagnostic guidelines for Alzheimer’s disease. Alzheimers Dement. 2011;7(3):263. 21514250 10.1016/j.jalz.2011.03.005 PMC3312024

[19] Nummenmaa L, Glerean E, Hari R, Hietanen JK. Bodily maps of emotions. Proc Natl Acad Sci U S A. 2014;111(2):646–51. doi: 10.1073/pnas.1321664111 24379370 PMC3896150

[20] Zaki J, Weber J, Bolger N, Ochsner K. The neural bases of empathic accuracy. Proc Natl Acad Sci U S A. 2009;106(27):11382–7. doi: 10.1073/pnas.0902666106 19549849 PMC2708723

[21] Corcoran R, Mercer G, Frith CD. Schizophrenia, symptomatology and social inference: investigating “theory of mind” in people with schizophrenia. Schizophr Res. 1995;17(1):5–13. doi: 10.1016/0920-9964(95)00024-g 8541250

[22] Spek AA, Scholte EM, Van Berckelaer-Onnes IA. Theory of mind in adults with HFA and Asperger syndrome. J Autism Dev Disord. 2010;40(3):280–9. doi: 10.1007/s10803-009-0860-y 19763808

[23] Stone VE, Baron-Cohen S, Knight RT. Frontal lobe contributions to theory of mind. J Cogn Neurosci. 1998;10(5):640–56. doi: 10.1162/089892998562942 9802997

[24] Gregory C, Lough S, Stone V, Erzinclioglu S, Martin L, Baron-Cohen S. Theory of mind in patients with frontal variant frontotemporal dementia and Alzheimer’s disease: theoretical and practical implications. Brain. 2002;125(Pt 4):752–64. 11912109 10.1093/brain/awf079

[25] Dodich A, Cerami C, Canessa N, Crespi C, Iannaccone S, Marcone A, et al. A novel task assessing intention and emotion attribution: Italian standardization and normative data of the Story-based Empathy Task. Neurol Sci. 2015;36(10):1907–12. doi: 10.1007/s10072-015-2281-3 26072203

[26] Happé F, Brownell H, Winner E. Acquired “theory of mind” impairments following stroke. Cognition. 1999;70(3):211–40. 10384736 10.1016/s0010-0277(99)00005-0

[27] van den Berg E, Poos JM, Jiskoot LC, Montagne B, Kessels RPC, Franzen S, et al. Impaired Knowledge of Social Norms in Dementia and Psychiatric Disorders: Validation of the Social Norms Questionnaire-Dutch Version (SNQ-NL). Assessment. 2022;29(6):1236–47. doi: 10.1177/10731911211008234 33855860 PMC9301163

[28] Faul F, Erdfelder E, Buchner A, Lang A-G. Statistical power analyses using G*Power 3.1: tests for correlation and regression analyses. Behav Res Methods. 2009;41(4):1149–60. doi: 10.3758/BRM.41.4.1149 19897823

